# Kidney function, uric acid, and risk of atrial fibrillation: experience from the AMORIS cohort

**DOI:** 10.1186/s12872-024-04236-9

**Published:** 2024-10-22

**Authors:** Mozhu Ding, Katharina Schmidt-Mende, Juan-Jesus Carrero, Gunnar Engström, Niklas Hammar, Karin Modig

**Affiliations:** 1 Unit of Epidemiology, Institute of Environmental Medicine, Karolinska Institutet, Nobelsväg 13, Stockholm, 17177 Sweden; 2 Academic Primary Health Care Centre, Stockholm Region, Stockholm, Sweden; 3 Division of Family Medicine and Primary Care, Department of Neurobiology, Care Sciences and Society, Karolinska Institutet, Huddinge, Sweden; 4 Department of Medical Epidemiology and Biostatistics, Karolinska Institutet, Stockholm, Sweden; 5 Division of Nephrology, Danderyd Hospital, Department of Clinical Sciences, Karolinska Institutet, Stockholm, Sweden; 6 Department of Clinical Sciences, Lund University, Malmö, Sweden

**Keywords:** Atrial fibrillation, Kidney function, Cohort study, Uric acid, Hyperuricemia

## Abstract

**Background:**

Uric acid closely relates to both kidney disease and atrial fibrillation (AF), yet the extent to which it influences the kidney-AF association remains uncertain. We examined the relationship between kidney function and risk of AF, accounting for uric acid levels.

**Methods:**

A total of 308,509 individuals in the Swedish Apolipoprotein-Related Mortality Risk (AMORIS) cohort were included and their serum creatinine and uric acid were measured during 1985–1996. Ten-year incident AF was identified via linkage with the national registers. Glomerular filtration rate (eGFR) (ml/min/1.73 m^2^) was calculated with the 2009 Chronic Kidney Disease Epidemiology Collaboration equation. Hyperuricemia was defined as > 420 µmol/L for men and > 360 µmol/L for women.

**Results:**

Over a mean follow-up of 9.4 years, 10,007 (3.2%) incident AF cases occurred. After adjusting for age, sex, cardiovascular diseases, total cholesterol, triglycerides, and glucose, individuals with low eGFR (< 30 and 30–59 ml/min/1.73 m^2^ ) had a higher risk of AF compared to those with normal eGFR (60–89) (hazard ratio (HR) = 1.72, 95% confidence interval (CI):1.29–2.30; HR = 1.10, 95% CI: 1.03–1.18, respectively). After further adjusting for uric acid levels, the association disappeared (HR = 0.97, 95% CI: 0.72–1.30; HR = 0.93, 95% CI: 0.86-1.00, respectively). When stratifying by hyperuricemia yes/no, eGFR < 30 ml/min/1.73 m^2^ was associated with higher AF risk in a small group of individuals without hyperuricemia (HR = 2.58, 95% CI: 1.64–4.07).

**Conclusion:**

Uric acid largely accounted for the relationship between eGFR and AF in this study. However, in individuals without hyperuricemia, eGFR in the lowest range (< 30 ml/min/1.73 m^2^) was still associated with increased risk of AF.

**Supplementary Information:**

The online version contains supplementary material available at 10.1186/s12872-024-04236-9.

## Introduction

Atrial fibrillation (AF) is the most common sustained cardiac arrhythmia affecting 38 million individuals worldwide [1]. AF is a major public health concern as it has a lifetime risk of around 30% after age 55 and is associated with elevated risk of ischemic stroke, cardiovascular diseases (CVDs), and mortality [2]. 

Known risk factors for AF include advanced age, male sex, hypertension, diabetes, heart failure, and lifestyle factors [3, 4]. Recently, kidney dysfunction has also been studied in relation to AF. It is a common clinical observation that kidney disease and AF coexist among patients [5–8]. However, whether kidney dysfunction independently increases the risk of AF is less clear. A few cohort studies reported a higher incidence of AF among individuals with reduced kidney function after adjusting for shared risk factors [9–12], while others don’t [13, 14]. Furthermore, in two recent Mendelian Randomization (MR) analyses, reduced kidney function measured by estimated glomerular filtration rate (eGFR) did not appear to be a causal risk factor for AF [15, 16]. Inconsistency in past evidence could be the result of different populations studied and different measures of kidney function used. Moreover, the diverging results from observational and MR studies indicate that the kidney-AF association may be, at least partly, explained by a third factor that has not been previously examined but closely intertwines with both.

Over the past decade, uric acid has gained renewed interest due to its increasingly recognized role in both CVDs and chronic kidney disease [17–19]. Uric acid is the end-product of purine metabolism in humans and is largely excreted by the kidney. Hyperuricemia is highly prevalent among individuals with reduced kidney function and has also emerged as a strong risk factor for cardiometabolic diseases including AF [17, 18, 20]. However, whether and how uric acid affects the association between kidney function and risk of AF has not been studied. This is an important area to investigate if we want to elucidate this cardio-renal relationship as well as modifiable risk factors for AF. To this end, this study sought to assess the association between kidney function and risk of AF in a large Swedish population-based cohort, taking into account uric acid levels.

## Methods

### Study population

We used data from the large population-based AMORIS (Apolipoprotein-Mortality Risk) cohort which has been developed to assess the role of metabolic factors and inflammation in chronic diseases. The cohort included 812,073 Swedish individuals with health examinations and blood measurements from 1985 to 1996. These individuals had their blood tests taken either as part of a general health check-up of healthy subjects in the occupational setting, or health examinations within primary or occupational health care. All laboratory tests were conducted on fresh blood samples by the Central Automation Laboratory (CALAB), Stockholm, Sweden. Several clinically commonly assessed biomarkers (e.g., total cholesterol, triglycerides, glucose, uric acid, and serum creatinine) were included in a standard analysis package offered by CALAB, and these biomarkers were available for a large proportion of the cohort.

A total of 313,698 individuals in the AMORIS cohort had measurements on serum creatinine, uric acid, total cholesterol, triglycerides, and glucose on the same day, and were aged ≥ 40 years at the time of blood measurements (baseline). Of these, we excluded 1113 individuals who migrated into Sweden, 1166 who had missing information on migration in the Total Population Register, 48 who died on the day of blood sampling, and 2862 who had a history of AF diagnosis at the time of blood measurement. The analytical sample eventually consisted of 308,509 individuals. These individuals were followed up via linkage with multiple national registers including the Swedish National Patient Register, Cause of Death Register, and Total Population Register using a unique Swedish personal identification number for each person. All individuals were followed from the day of blood measurements over a maximum of 10 years for incident AF diagnosis, migration, deaths, or end of follow-up. The follow-up period was limited to 10 years because plasma creatinine and uric acid could vary over long study periods and result in misclassification of exposure.

This study complies with the 1957 Declaration of Helsinki and has been approved by the regional ethical committee at Karolinska Institutet, Stockholm, Sweden (reference number 2018/2401–31). The ethical board waived the need for informed consent in the AMORIS cohort.

## Assessment of kidney function, uric acid, and other biomarkers

Serum creatinine was analyzed by a non-kinetic alkaline picrate method (Jaffe Method) using an AutoChemist-PRISMA during 1985–1992 and DAX-96 analyzer during 1993–1996. The coefficients of variation for creatinine determinations were less than 3.1% at 75.5 µmol/L, 1.7% at 212 µmol/L and 1.6% at 547 µmol/L. The 2009 Chronic Kidney Disease Epidemiology Collaboration (CKD-EPI) formula was used to estimate eGFR based on serum creatinine [21]. Because at the time of AMORIS blood sampling creatinine was measured with a non-isotope dilution mass spectroscopy-traceable method, crude creatinine values were reduced by 5% before being entered into the CKD-EPI formula [22–24]. eGFR is categorized according to the recommendations from the Kidney Disease Improving Global Outcomes (KDIGO) initiative: <30 (severe dysfunction or kidney failure), 30–59 (mild to moderate dysfunction), 60–89 (mild dysfunction to normal), and ≥ 90 (normal to high) mL/min/1.73 m^2^.

Serum uric acid (µmol/L) was measured by the enzymatic uricase method. Variation for uric acid determinations were < 2.8% at 164 µmol/L (2.76 mg/dL), 2.3% at 470 µmol/L (7.90 mg/dL), and 1.8% at 624 µmol/L (10.49 mg/dL). Hyperuricemia is defined as > 420 µmol/L for men and > 360 µmol/L for women [25]. Covariates included sex (men/women), age (years), blood glucose (mmol/L), total cholesterol (mmol/L), and triglycerides (mmol/L).

## Ascertainment of atrial fibrillation and other conditions

Incident AF cases developed during the follow-up were identified from the Swedish National Patient Register and the Cause of Death Register. The Swedish National Patient Register contains information on hospital discharge records from inpatient care regionally since 1964 and nationally since 1987, and data on specialized outpatient care were available nationally since 2001. Data in this register includes the dates and discharge diagnoses of each hospital visit, and all diagnoses were coded according to the International Classification of Diseases, Eighth Revision, Ninth Revision, and Tenth Revision (ICD-8, ICD-9, ICD-10). The National Cause of Death Register is a complete register of all deaths in Sweden since 1952, with ICD codes of underlying and contributing causes of deaths. In this study, incident AF was identified as the first diagnosis appearing in either the National Patient Register or the Cause of Death Register (ICD-8: 427.90 and 427.92; ICD-9: 427.3; ICD-10: I48).

Other cardiovascular conditions prior to baseline were identified from the Swedish National Patient Register and include diabetes (ICD-8: 250; ICD-9: 250, 251.D; ICD-10: E10, E11, E13, E14), hypertension (ICD-8: 400–404; ICD-9: 401–405; ICD-10: I10, I13, I15), heart failure (ICD-8: 427.0, 427.1; ICD-9: 402, 404, 425, 428; ICD-10: I110, I130, I132, I27, I280, I42, I43, I50, I515, I517, I528), coronary heart disease (ICD-8: 410–414; ICD-9: 410–414; ICD-10: I20-I25), stroke (ICD-8: 431–434; ICD-9: 431–434; ICD-10: I61, I63, I64), and transient ischemic attack (TIA) (ICD-8: 435; ICD-9: 435; ICD-10: G45).

### Statistical analysis

Cox proportional hazard models with restricted cubic splines were used to assess the association between continuous eGFR and incident AF. The eGFR value with the lowest AF risk (i.e., 75 mL/min/1.73 m^2^) was set as the reference point. Then, we studied eGFR categories in relation to incident AF in Cox regression models, with 60–89 mL/min/1.73 m^2^ as the reference group. In both spline and categorical analyses, the models were first adjusted for age, sex, CVDs at baseline (i.e., hypertension, diabetes, coronary heart disease, heart failure, stroke, and TIA), and serum concentrations of total cholesterol, triglycerides, and glucose. Then, uric acid was entered as a continuous variable to the above models to investigate whether and to what extend the associations between eGFR and AF change. Moreover, we performed stratified analyses to explore the association between eGFR and AF in the presence or absence of hyperuricemia.

All statistical analyses were conducted using Stata 16.1 (StataCorp, College Station, TX). Effect estimates were presented as hazard ratio (HR) and 95% confidence interval (CI).

Clinical trial number: not applicable.

## Results

Mean age at baseline was 53.4 years. A total of 304 individuals (0.1%) were categorized as eGFR < 30 ml/min/1.73 m^2^, 10,676 (3.5%) as 30–59 ml/min/1.73 m^2^, 150,772 (48.9%) as 60–89 ml/min/1.73 m^2^, and 146,757 (47.6%) as ≥ 90 ml/min/1.73 m^2^. Individuals with lower eGFR were older, more likely to be women, had more CVDs, and had higher concentrations of total cholesterol, glucose, and triglycerides (Table 1). More than 63% of individuals with eGFR < 30 ml/min/1.73 m^2^ were categorized as having hyperuricemia, while only 8.5% of individuals with eGFR 60–89 ml/min/1.73 m^2^ had hyperuricemia. Mean uric acid levels were substantially higher among individuals with eGFR < 30 and 30–59 as compared to those with 60–89 and ≥ 90 ml/min/1.73 m^2^ (Supplemental Fig. 1).

**Table 1:** Characteristics of the study population by eGFR categories

Baseline characteristics	Total	eGFR categories (ml/min/1.73 m^2^)			
		< 30	30–59	60–89	≥ 90
No. of subjects, n (%)	308,509	304 (0.1)	10,676 (3.5)	150,772 (48.9)	146,757 (47.6)
Age (years), mean (SD)	53.4 (10.1)	70.8 (13.7)	70.8 (10.9)	56.4 (10.2)	49.0 (6.8)
Age groups (years), n (%)					
40–65	271,053 (87.9)	97 (31.9)	3363 (31.5)	123,389 (81.8)	144,204 (98.2)
65–74	24,353 (7.9)	71 (23.4)	2949 (27.6)	18,870 (12.5)	2463 (1.7)
≥ 75	13,103 (4.3)	136 (44.7)	4364 (40.9)	8513 (5.7)	90 (0.1)
Women, n (%)	146,176 (47.4)	158 (52.0)	7811 (73.2)	84,102 (55.8)	54,105 (36.9)
Hospital diagnosis of comorbidities, n (%)					
Hypertension	4251 (1.4)	65 (21.4)	647 (6.1)	2396 (1.6)	1143 (0.8)
Diabetes	2814 (0.9)	38 (12.5)	351 (3.3)	1351 (0.9)	1074 (0.7)
Heart failure	1418 (0.5)	47 (15.5)	455 (4.3)	741 (0.5)	175 (0.1)
Coronary heart disease	6767 (2.2)	54 (17.8)	985 (9.2)	4073 (2.7)	1655 (1.1)
Stroke/transient ischemic attack	2226 (0.7)	29 (9.5)	355 (3.3)	1298 (0.9)	544 (0.4)
Hyperuricemia, n (%)	27,658 (9.0)	194 (63.8)	3563 (33.4)	12,767 (8.5)	6551 (4.5)
Metabolic biomarkers					
Uric acid (µmol/L), mean (SD)	294.2 (74.4)	449.1 (131.1)	349.6 (91.8)	297.1 (73.6)	286.8 (71.3)
Total cholesterol (mmol/L), mean (SD)	5.9 (1.1)	6.1 (1.6)	6.3 (1.3)	6.0 (1.1)	5.8 (1.1)
Glucose (mmol/L), mean (SD)	5.1 (1.4)	6.0 (2.7)	5.6 (2.2)	5.2 (1.5)	5.1 (1.2)
Triglycerides (mmol/L), mean (SD)	1.4 (1.0)	2.3 (1.8)	1.7 (1.1)	1.4 (1.0)	1.4 (1.1)

SD = standard deviation, eGFR = estimated glomerular filtration rate

**Fig. 1:**
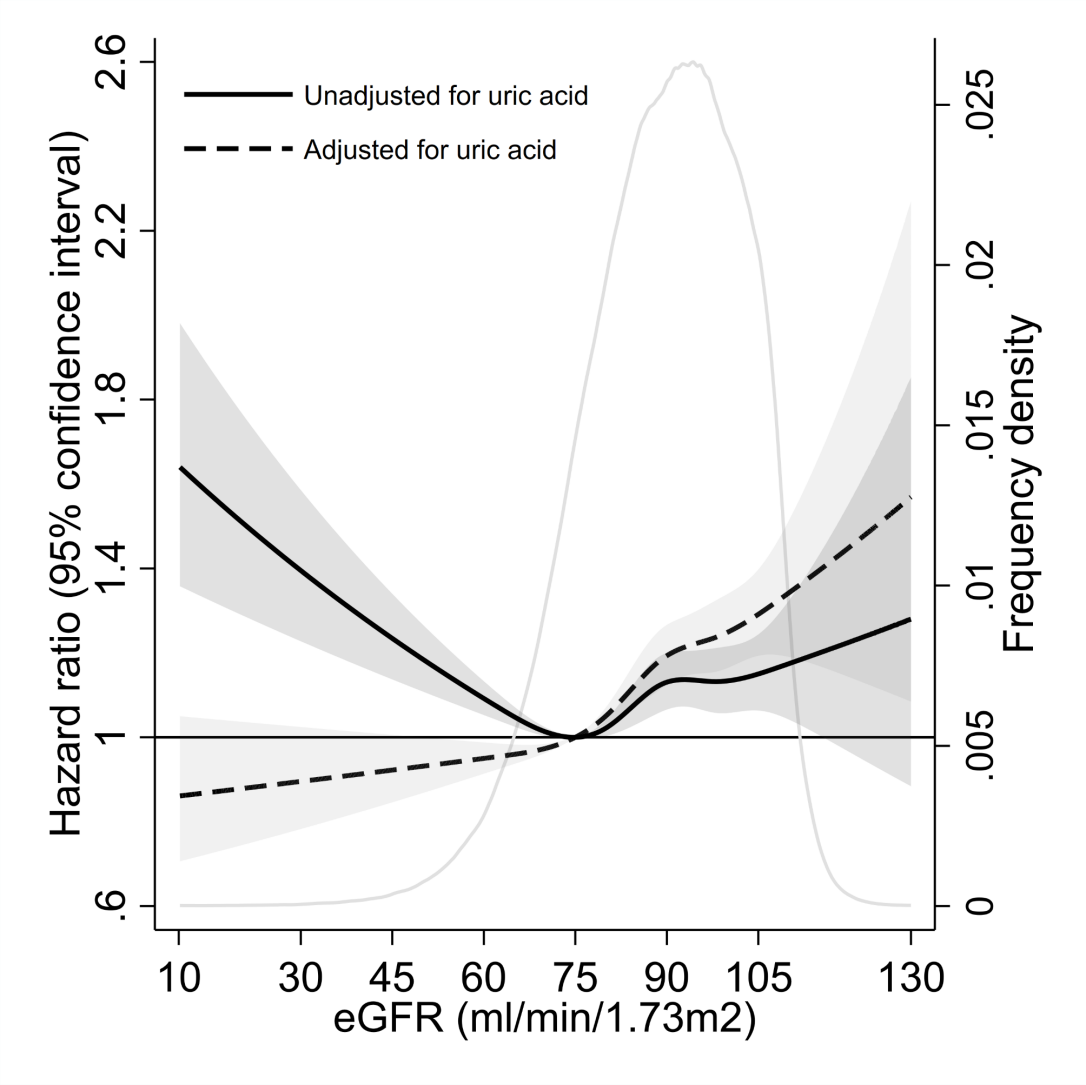
Hazard ratios for atrial fibrillation associated with continuous eGFR. Black solid line represents hazard ratios adjusted for age, sex, coronary heart disease, heart failure, stroke/transient ischemic attack, hypertension, diabetes, triglycerides, total cholesterol, and glucose. Black dashed line represents hazard ratios further adjusted for uric acid levels. Gray areas are 95% confidence intervals. Gray solid line indicates the distribution of eGFR in the study population. eGFR = estimated glomerular filtration rate

Over the 10-year follow-up (mean 9.4 years, SD 1.9), 10,007 (3.2%) incident AF cases occurred. Cox regression model with restricted cubic splines showed a reverse J-shape association between continuous eGFR and incident AF, after adjusting for age, sex, coronary heart disease, heart failure, stroke/transient ischemic attack, hypertension, diabetes, triglycerides, total cholesterol, and glucose (Fig. 1). eGFR 75 ml/min/1.73 m^2^ was associated with the lowest AF risk in the multi-adjusted model. Although eGFR lower and higher than 75 were both associated with an elevated AF risk, the increased risk was much more pronounced for eGFR lower than 75. However, when further adjusting for uric acid concentrations, the association between eGFR < 75 ml/min/1.73 m^2^ and risk of AF was largely attenuated. On the other hand, continuous uric acid showed a strong linear and dose-response association with incident AF after adjusting for age, sex, CVDs, triglycerides, total cholesterol, glucose, and eGFR (Supplemental Fig. 2).

When eGFR was analyzed based on the KDIGO categorization, results were similar to those from the spline analyses. eGFR < 30 and 30–59 ml/min/1.73 m^2^ was statistically significantly associated with an increased AF risk after adjusting for age, sex, CVDs, and metabolic markers, compared to eGFR 60–89 ml/min/1.73 m^2^ (HR = 1.72, 95% CI:1.29–2.30; HR = 1.10, 95% CI: 1.03–1.18, respectively) (Table 2). However, when further adjusting for uric acid levels, the association of eGFR < 30 and 30–59 ml/min/1.73 m^2^ with incident AF disappeared (HR = 0.97, 95% CI: 0.72–1.30; HR = 0.93, 95% CI: 0.86-1.00, respectively). eGFR ≥ 90 ml/min/1.73 m^2^ was associated with a slight increase in AF risk in the fully adjusted model (HR = 1.17, 95% CI: 1.11–1.23). Similar patterns were also observed in the sex-stratified analyses (Supplemental Table 1). With regards to absolute risk, incidence rate (IR) of AF was highest among individuals with eGFR < 30 (IR = 30.4 per 1000 person-years, 95% CI: 22.8–40.5) and lowest among those with eGFR ≥ 90 ml/min/1.73 m^2^ (IR = 2.0 per 1000 person-years, 95% CI: 1.9-2.0) (Table 2).

**Table 2:** Incidence rate and hazard ratios (95% confidence interval) for 10-year incident atrial fibrillation associated with estimated glomerular filtration rate categories

eGFR categories (ml/min/1.73 m^2^)	No. of subjects	No. of new AF (%)	IR per 1000 PY (95% CI)	Hazard ratios (95% confidence interval)		
				Model 1 (age + sex)	Model 2 (Model 1 + CVDs + biomarkers)	Model 3 (Model 2 + uric acid)
< 30	304	47 (15.5)	30.4 (22.8–40.5)	2.52 (1.90–3.37)^a^	1.72 (1.29–2.30)^a^	0.97 (0.72–1.30)
30–59	10,676	1260 (11.8)	15.3 (14.5–16.1)	1.19 (1.12–1.28)^a^	1.10 (1.03–1.18)^a^	0.93 (0.86-1.00)
60–89	150,772	5954 (4.0)	4.2 (4.1–4.3)	Reference (1.00)	Reference (1.00)	Reference (1.00)
≥ 90	146,757	2746 (1.9)	2.0 (1.9-2.0)	1.08 (1.02–1.14)^a^	1.09 (1.03–1.15)^a^	1.17 (1.11–1.23)^a^

Model 1: age and sex; Model 2: Model 1 + coronary heart disease, heart failure, stroke/transient ischemic attack, hypertension, diabetes, and levels of triglycerides, total cholesterol, and glucose; Model 3: Model 2 + uric acid levels. AF = atrial fibrillation; eGFR = estimated glomerular filtration rate; CI = confidence interval; CVD = cardiovascular disease. IR per 1000 PY = incidence rate per 1000 person-years. ^a^*p*<0.05

Table 3 shows the stratified analyses by hyperuricemia status. Either with or without hyperuricemia, eGFR between 30 and 59 was not associated with incident AF compared to eGFR 60–89 ml/min/1.73 m^2^ in the multi-adjusted model (HR = 1.02, 95% CI: 0.90–1.16, HR = 0.93, 95% CI: 0.86–1.02, respectively). eGFR < 30 ml/min/1.73 m^2^ was not associated with incident AF among individuals with hyperuricemia (HR = 0.83, 95% CI: 0.56–1.25), but was associated with an elevated risk of AF among those without hyperuricemia (HR = 2.58, 95% CI: 1.64–4.07).

**Table 3:** Incidence rate and hazard ratios (95% confidence interval) for 10-year incident atrial fibrillation associated with estimated glomerular filtration rate categories, stratified by hyperuricemia status

eGFR categories (ml/min/1.73 m^2^)	No. of subjects	No. of new AF (%)	IR per 1000 PY (95% CI)	Hazard ratio (95% confidence interval)		
				Model 1 (age + sex)	Model 2 (Model 1 + CVDs + biomarkers)	Model 3 (Model 2 + uric acid)
Among individuals with hyperuricemia						
< 30	194	28	28.6 (19.7–41.3)	1.47 (1.00-2.16)^a^	1.22 (0.82–1.79)	0.83 (0.56–1.25)
30–59	3563	539	21.4 (19.6–23.2)	1.11 (0.98–1.26)	1.09 (0.96–1.23)	1.02 (0.90–1.16)
60–89	12,767	881	7.7 (7.2–8.2)	Reference (1.00)	Reference (1.00)	Reference (1.00)
≥ 90	6551	248	4.1 (3.5–4.6)	1.12 (0.96–1.30)	1.11 (0.96–1.30)	1.12 (0.96–1.31)
Among individuals without hyperuricemia						
< 30	110	19	33.7 (21.5–52.9)	3.70 (2.35–5.81)^a^	2.80 (1.78–4.41)^a^	2.58 (1.64–4.07)^a^
30–59	7113	721	12.6 (11.7–13.5)	1.05 (0.97–1.14)	1.00 (0.93–1.10)	0.93 (0.86–1.02)
60–89	138,005	5073	3.9 (3.8-4.0)	Reference (1.00)	Reference (1.00)	Reference (1.00)
≥ 90	140,206	2498	1.9 (1.7–1.9)	1.13 (1.08–1.20)^a^	1.13 (1.07–1.19)^a^	1.19 (1.12–1.25)^a^

Model 1: age and sex; Model 2: Model 1 + coronary heart disease, heart failure, stroke/transient ischemic attack, hypertension, diabetes, and levels of triglycerides, total cholesterol, and glucose; Model 3: Model 2 + continuous uric acid levels. AF = atrial fibrillation; eGFR = estimated glomerular filtration rate; CI = confidence interval; HR = hazard ratio; IR per 1000 PY = incidence rate per 1000 person-years. ^a^*p*<0.05

## Discussion

This large population-based study explored whether and how uric acid levels affects the association between kidney function and incident AF. We found that (1) the association between kidney dysfunction (eGFR < 60 ml/min/1.73 m^2^) and higher risk of AF disappeared when accounting for uric acid levels, (2) in stratified analyses, when hyperuricemia was present, low kidney function was not associated with AF. However, when hyperuricemia was absent, severe kidney dysfunction (eGFR < 30 ml/min/1.73 m^2^) remained associated with higher risk of AF. These results suggest that, overall, uric acid plays an important role in the association between kidney dysfunction and higher AF risk. However, there is a subgroup of patients with severe kidney dysfunction but normal uric acid level for whom AF risk is consistently elevated.

Previous observational studies have reported mixed results on the association between low eGFR and incident AF, where some support an association [9, 12] and others don’t [13, 14]. Yet no previous studies have accounted for uric acid when analyzing kidney function and AF, despite that it closely relates to both. MR studies using genetically predicted eGFR did not find evidence for a causal role on AF [15, 16, 26], and neither on heart failure [26, 27], a cardiovascular condition that often co-occurs with AF. However, previous MR analyses mostly tested the effect of mild-to-moderate kidney dysfunction on AF, and the cardiovascular effects from severe kidney dysfunction or kidney failure remains unknown [16]. In this study we further show that uric acid largely accounts for the association between mild-to-moderate kidney dysfunction and AF, but less consistently so for severe kidney dysfunction.

It is very difficult to sort out whether low kidney function is caused by or is the result of high uric acid levels as the two interact [18, 28]. It is thus unclear in our analyses whether uric acid is a mediator or a confounder, or likely both. In chronic kidney disease, serum uric acid levels rise due to reductions in eGFR. Elevated uric acid can also lead to kidney disease by causing endothelial dysfunction, activation of the renin-angiotensin-aldosterone system (RAAS), inflammation, and oxidative stress [18, 28]. Interestingly, elevated uric acid has been shown to promote the onset of AF through similar mechanisms including RAAS activation and oxidative stress.^28^ This overlap in mechanisms may to some extent explain why in our study the association between low eGFR and higher risk of AF disappeared after accounting for continuous uric acid. Moreover, we show that, when hyperuricemia is present, any stage of low kidney function (mild-to-moderate or severe) was not associated with future risk of AF in the multi-adjusted model. This suggests that uric acid might be more of a driving factor for AF onset than kidney dysfunction per se when uric acid is clearly elevated. Uric acid is the end-product of purine metabolism; its serum concentration is related to various sources including purine-rich food, alcohol consumption, fructose metabolism, gut microbiota, and medications (e.g., diuretics). As we lack lifestyle and medication data at the time of blood measurement, we could not further investigate how these factors could have affected uric acid levels in our study. Uric acid levels are in general more modifiable than kidney function, which is difficult to reverse once it starts to decline, especially in old age. Future studies are needed to investigate whether treating hyperuricemia, either through lifestyle modification or medications, could lower the risk of AF among individuals with impaired kidney dysfunction. Existing data have shown that individuals with early-stage kidney disease benefit more from cardiovascular risk factor management in reducing future cardiovascular events than those in end-stage [30]. 

For individuals with severe kidney dysfunction (eGFR < 30), more than half (63.8%) had hyperuricemia, consistent with previous studies [31, 32]. However, not all individuals with kidney dysfunction present with hyperuricemia. In our study, 36.2% of individuals with severe kidney dysfunction were free of hyperuricemia (*n* = 110), a small fraction of the total population. Yet we found an elevated risk of AF with an HR of 2.58 for this small subgroup even after accounting for continues uric acid levels. It is possible that uric acid needs to surpass the threshold of hyperuricemia in order to have an active role from kidney disease to cardiovascular risk [18]. This might be the reason why in our study uric acid levels did not explain away the association between severe kidney dysfunction and AF in people with normal uric acid levels. Our results instead suggest that without hyperuricemia, severe kidney dysfunction has a strong and independent association with AF.

Moreover, it should be noted that studies using other measures of kidney dysfunction such as urinary albumin excretion or albuminuria have more consistently reported an association with increased AF risk, both from cohort studies [33–35] and MR analyses [15]. Both eGFR and albumin excretion are markers of kidney dysfunction. However, eGFR is more a marker of kidney disease per se, and urinary albumin is more a marker of systemic vascular damage or microvascular disease [36]. Even though we did not find an independent association between low kidney dysfunction and AF in the full sample analyses, our results do not rule out the possibility that microvascular damage associated with low kidney dysfunction could still promote AF onset.

We also found that eGFR ≥ 90 ml/min/1.73 m^2^ is associated with a slight increased risk of AF after multiple adjustments. This should be interpreted with caution as eGFR is less predictive of cardiovascular risk in predominantly healthier populations with normal renal function, and more predictive in populations with chronic kidney disease [37, 38]. eGFR also tends to overestimate kidney function in individuals with low muscle mass, low body size, or diseases that result in muscle-catabolism and cachexia [39], potentially explaining the slight increase in AF risk for eGFR ≥ 90 ml/min/1.73 m^2^ in our study. However, it should be noted that the incidence rate of AF is still the lowest among individuals with eGFR ≥ 90 ml/min/1.73 m^2^ in this study. Yet eGFR 60–89 ml/min/1.73 m^2^ was chosen as the reference group because the eGFR value that has the lowest AF risk (i.e., 75 ml/min/1.73 m^2^) in the spline analyses falls within this range. This is also consistent with prior large meta-analyses pooling data from 21 prospective cohorts in which all-cause mortality and cardiovascular mortality decreases until eGFR 75 ml/min/1.73 m^2^ and remained relatively stable from 75 and onwards [38]. 

A few limitations should be considered. First, as this is an observational study, we cannot draw conclusions on causal inference. Even though we report attenuated association between low eGFR and AF after multiple adjustments, residual confounding is still possible, and we acknowledge the lack of data on medication use and lifestyle factors such as body mass index and alcohol consumption. Moreover, as we restricted the follow-up time to 10 years after blood measurements, incident AF cases were identified up to the end of 2005. Because outpatient data were only available from 2001 and onwards, it is likely that we missed some AF diagnoses in the first few years of follow up, which could have slightly diluted the association between low eGFR and incident AF. Given that primary care data is not available in our study, we could also have missed cases that are diagnosed only in primary care settings including AF, hypertension, diabetes, and heart failure. However, we adjusted for continuous glucose levels in the models which could account for some prediabetes and diabetes cases. Finally, we only had one measurement of eGFR and uric acid at baseline and we do not know the time order of hyperuricemia and low kidney function.

## Conclusions

Overall, uric acid plays an important role in the kidney-AF association. In the presence of hyperuricemia, low kidney function did not in itself appear to elevate the risk of AF. Yet in individuals with normal uric acid levels, severe kidney dysfunction has a strong and independent association with AF; this group is however very small. More research is needed to confirm our findings, and to explore the underlying mechanisms. Moreover, evidence is needed to show whether treating hyperuricemia among individuals with impaired kidney function could lower the risk of future AF.

## Electronic supplementary material

Below is the link to the electronic supplementary material.

Supplementary Material 1

## Data Availability

Due to the General Data Protection Regulation in Sweden, the pseudo-anonymized personal data underlying this study cannot be shared publicly. Access to the data and the codes for data analyses can be permitted to external researchers after ethical vetting and establishment of a collaboration agreement. Contact the corresponding author for questions about data sharing (MD).
