# Supplementary material for: Kidney function, uric acid, and risk of atrial fibrillation: experience from the AMORIS cohort
Source: BMC Cardiovasc Disord. 2024 Oct 22;24:581. doi: 10.1186/s12872-024-04236-9 (PMC11494868; doi:10.1186/s12872-024-04236-9)
Supplement: Supplementary file 1 — Supplementary Material 1 [file 12872_2024_4236_MOESM1_ESM.docx]

**Supplemental Table 1**. Incidence rate and hazard ratios (95% confidence interval) for 10-year incident atrial fibrillation associated with eGFR categories, stratified by sex.

| **eGFR categories (ml/min/1.73m^2^)** | No. of subjects | No. of new AF (%) | IR per 1000 PY (95% CI) | Hazard ratios (95% confidence interval) | | |
| --- | --- | --- | --- | --- | --- | --- |
|  |  |  |  | Model 1 (age + sex) | Model 2 (Model 1 + CVDs + biomarkers) | Model 3 (Model 2 + uric acid) |
| **Men** |  |  |  |  |  |  |
| <30 | 146 | 15 (10.3) | 19.7 (11.9-32.6) | 1.82 (1.10-3.03)^a^ | 1.37 (0.82-2.28) | 0.87 (0.52-1.45) |
| 30-59 | 2865 | 431 (15.0) | 21.5 (19.6-23.7) | 1.37 (1.23-1.52)^a^ | 1.23 (1.10-1.36)^a^ | 1.01 (0.90-1.13) |
| 60-89 | 66670 | 3287 (4.9) | 5.4 (5.2-5.6) | Reference (1.00) | Reference (1.00) | Reference (1.00) |
| ≥90 | 92652 | 2163 (2.3) | 2.5 (2.3-2.6) | 1.03 (0.97-1.10) | 1.04 (0.98-1.11) | 1.12 (1.06-1.20)^a^ |
| **Women** |  |  |  |  |  |  |
| <30 | 158 | 32 (20.3) | 40.9 (28.9-57.8) | 2.89 (2.03-4.10)^a^ | 1.82 (1.27-2.60)^a^ | 1.02 (0.70-1.48) |
| 30-59 | 7811 | 829 (10.6) | 13.3 (12.4-14.2) | 1.05 (0.96-1.14) | 0.98 (0.90-1.07) | 0.85 (0.78-0.93)^a^ |
| 60-89 | 84102 | 2667 (3.2) | 3.3 (3.2-3.5) | Reference (1.00) | Reference (1.00) | Reference (1.00) |
| ≥90 | 54105 | 583 (1.1) | 1.1 (1.0-1.2) | 1.10 (0.99-1.21) | 1.07 (0.90-1.19) | 1.14 (1.03-1.26)^a^ |

Model 1: age and sex; Model 2: Model 1 + coronary heart disease, heart failure, stroke/transient ischemic attack, hypertension, diabetes, and levels of triglycerides, total cholesterol, and glucose; Model 3: Model 2 + uric acid levels. AF=atrial fibrillation; eGFR= estimated glomerular filtration rate; CI=confidence interval; CVD=cardiovascular disease. IR per 1000 PY=incidence rate per 1000 person-years. ^a^p<0.05.


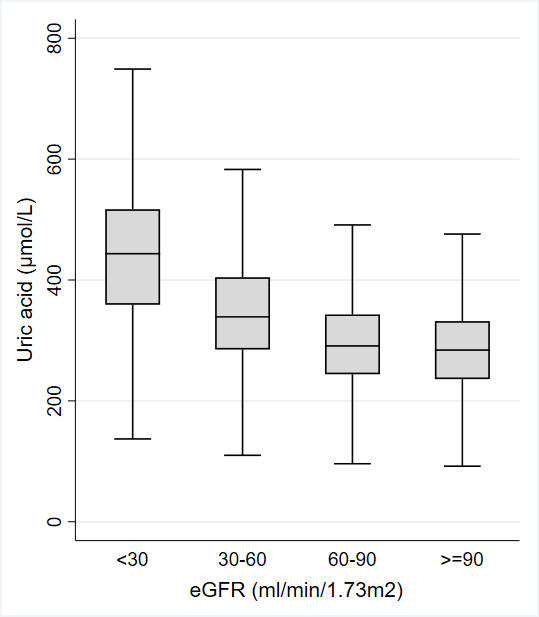


**Supplemental Figure 1.** Box plot of uric acid concentrations across categories of estimated glomerular filtration rate (eGFR).


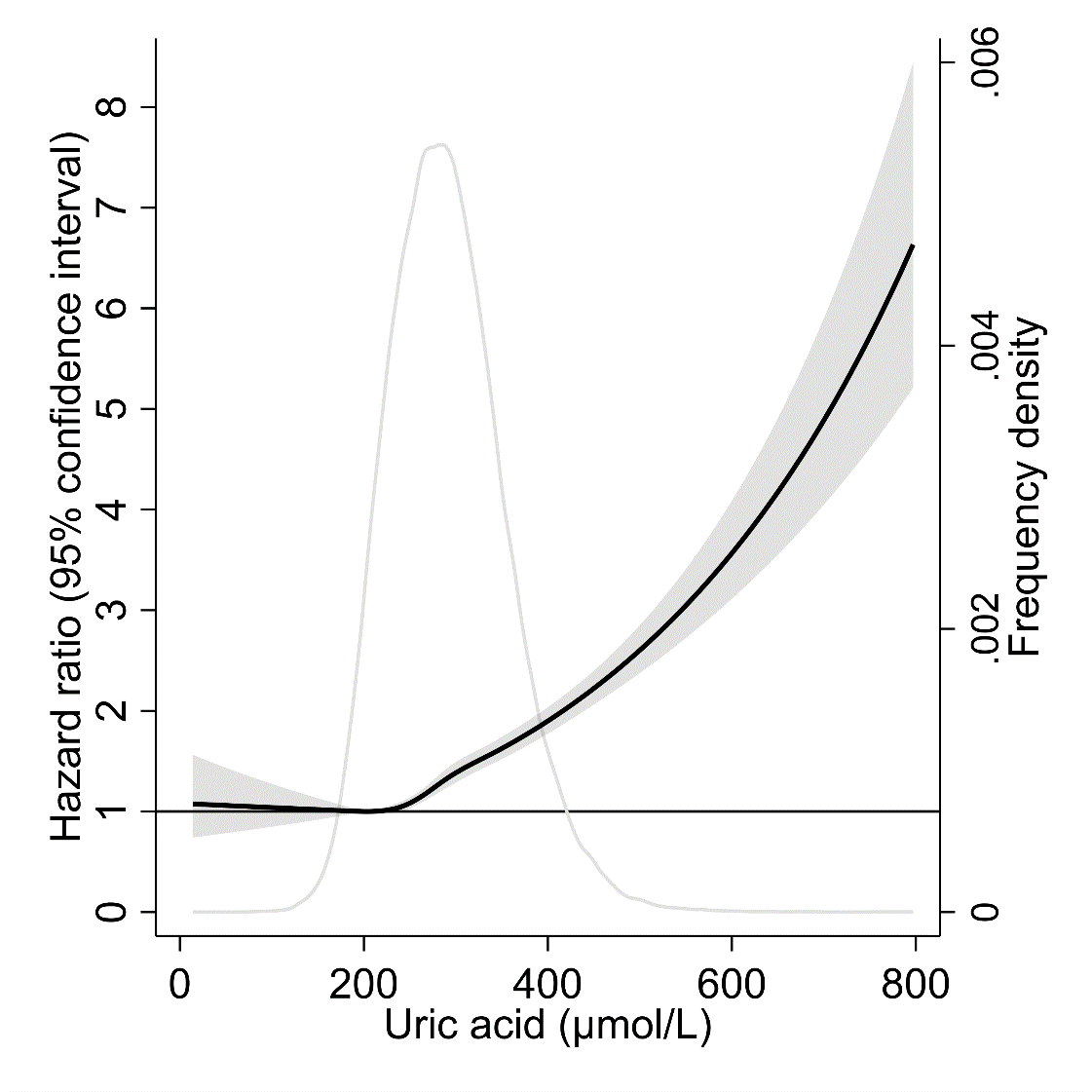


**Supplemental Figure 2**. Hazard ratios and 95% confidence interval for incident atrial fibrillation associated with continuous uric acid levels from Cox regression models with restricted cubic splines. Black solid line represents hazard ratios adjusted for age, sex, coronary heart disease, heart failure, stroke/transient ischemic attack, hypertension, diabetes, estimated glomerular filtration rate, triglycerides, total cholesterol, and glucose. Gray areas are 95% confidence intervals. Gray solid line indicates the distribution of uric acid in the study population.
